# Targeting TNFR2 as a Novel Therapeutic Strategy for Alzheimer’s Disease

**DOI:** 10.3389/fnins.2019.00049

**Published:** 2019-02-04

**Authors:** Natalia Ortí-Casañ, Yingying Wu, Petrus J. W. Naudé, Peter P. De Deyn, Inge S. Zuhorn, Ulrich L. M. Eisel

**Affiliations:** ^1^Department of Molecular Neurobiology, Groningen Institute for Evolutionary Life Sciences, Faculty of Science and Engineering, University of Groningen, Groningen, Netherlands; ^2^Department of Neurology and Alzheimer Center, University of Groningen, University Medical Center Groningen, Groningen, Netherlands; ^3^Department of Biomedical Engineering, University of Groningen, University Medical Center Groningen, Groningen, Netherlands

**Keywords:** tumor necrosis factor, Alzheimer’s disease, neurodegeneration, neuroprotection, agonists, antagonists

## Abstract

Alzheimer’s disease (AD) is a progressive neurodegenerative disorder and the most common cause of dementia. Accumulating experimental evidence shows the important linkage between tumor necrosis factor-α (TNF) and AD, but the exact role of TNF in AD is still not completely understood. Although TNF-inhibitors are successfully used for treating several diseases, total inhibition of TNF can cause side effects, particularly in neurological diseases. This is attributed to the opposing roles of the two TNF receptors. TNF receptor 1 (TNFR1) predominantly mediates inflammatory and pro-apoptotic signaling pathways, whereas TNF receptor 2 (TNFR2) is neuroprotective and promotes tissue regeneration. Therefore, the specific activation of TNFR2 signaling, either by directly targeting TNFR2 via TNFR2 agonists or by blocking TNFR1 signaling with TNFR1-selective antagonists, seems a promising strategy for AD therapy. This mini-review discusses the involvement of TNFR2 and its signaling pathway in AD and outlines its potential application as therapeutic target. A better understanding of the function of TNFR2 may lead to the development of a treatment for AD.

## Introduction

The pleiotropic pro-inflammatory cytokine tumor necrosis factor-α (TNF), known as a member of the TNF superfamily of ligands, is a master regulator of the innate and adaptive immune system. TNF plays a vital role in the initiation and orchestration of inflammation and immunity (Aggarwal, 2003; Apostolaki et al., 2010; Fischer and Maier, 2015), and is potentially a key player in several neurodegenerative disorders, including Alzheimer’s disease (AD) (McAlpine and Tansey, 2008).

Alzheimer’s disease is an age-related neurodegenerative disorder associated with severe cognitive impairment such as memory deficits, deterioration of visuospatial skills and executive dysfunction. Its neuropathological hallmarks include both positive and negative features. Positive lesions consist of amyloid plaques, neurofibrillary tangles, neuropil threads, tau protein hyperphosphorylation, and glial activation. Negative lesions include losses of neurons, neuropil, and synaptic elements. Besides the well-known pathological characteristics mentioned above, neuroinflammation has been considered a major contributor to AD, in which TNF is an important mediator (McAlpine and Tansey, 2008).

Tumor necrosis factor is expressed as a 26-kDa monomeric type II transmembrane protein (tmTNF) that can be cleaved to release a 17-kDa soluble monomeric protein (solTNF) by the matrix metalloprotease TNF converting enzyme (TACE/ADAM17) (Aggarwal, 2000; Idriss and Naismith, 2000). TNF binds to two cognate receptors, the 55-kDa TNF receptor 1 (TNFR1, p55, TNFRSF1A) and the 75-kDa TNF receptor 2 (TNFR2, p75, TNFRSF1B), that mediate a variety of cellular responses. In general, TNFR1 predominantly exerts pro-inflammatory effects and TNFR2 is neuroprotective and promotes tissue homeostasis and regeneration. The identification of TNF expression around amyloidogenic plaques in human AD post-mortem brain tissue was the first indication of its possible involvement in AD (Dickson, 1997). At the protein level, elevated serum and plasma levels of TNF have been detected in patients with AD (Fillit et al., 1991; Bruunsgaard et al., 1999; Collins et al., 2000; Alvarez et al., 2007; Tan et al., 2007). Moreover, overproduction of inflammatory mediators in the AD brain occurs when microglia become chronically activated. It has been suggested that resident microglia fail to efficiently phagocytose amyloid beta (Aβ) because of elevated levels of proinflammatory cytokines, including TNF (Koenigsknecht-Talboo and Landreth, 2005). Besides, cell culture studies showed that TNF increased apoptosis of neurons treated with Aβ (Blasko et al., 1997) and affected Aβ production (Blasko et al., 1999, 2000). Furthermore, at the genetic level, three TNF polymorphisms were found to be associated with AD (Collins et al., 2000). For example, the TNF G308A promoter polymorphisms was found to increase the risk of AD in certain people (Kang et al., 2015; Wang, 2015). As TNFR2 activation has recently emerged as a potential therapeutic approach for neurodegeneration (Dong et al., 2016), we will here introduce TNFR2, focusing on the interrelation of TNFR2 and AD, and summarize a potential therapeutic strategy to specifically target TNFR2 in AD.

## TNFR2 Expression and Structure

Tumor necrosis factor receptor 1 is ubiquitously expressed in virtually all cell types and tissues, and its activation can be induced by either solTNF or tmTNF, with a preference for solTNF. TNFR2 is predominately expressed in cells of the immune system, especially regulatory T (T_reg_) cells, and by endothelial cells, and preferentially binds tmTNF (Grell, 1995; Grell et al., 1998). TNFR1 and TNFR2 are single-pass transmembrane glycoproteins with 28% homology mainly in the extracellular domain, that is comprised of four cysteine-rich motif (Locksley et al., 2001). However, the intracellular domains of the TNF receptors are largely unrelated, lacking homologous sequences (Lewis et al., 1991), suggesting that different signaling functions originate from the two distinct receptors. TNFR1 contains an intracellular death domain (DD) that binds TNFR1-associated death domain protein (TRADD) of Fas-associated death domain (FADD), primarily involved in signaling for cell death. While TNFR2 does not contain a cytoplasmic DD, it interacts with TNF-associated factor 2 (TRAF2), and mainly yields cell survival (Wajant et al., 2003). TNFR2 consists of 439 amino acids, with the first 235 amino acids forming the extracellular domain, 30 amino acids forming the transmembrane domain, and 174 amino acids forming the cytoplasmic domain with a TRAF2 binding site. TRAF2 can bind TRAF1, TRAF3, inhibitor of apoptosis protein 1 (cIAP1) and inhibitor of apoptosis protein 2 (cIAP2) (Rothe et al., 1994, 1995b).

## TNFR2 Signaling

**FIGURE 1:**
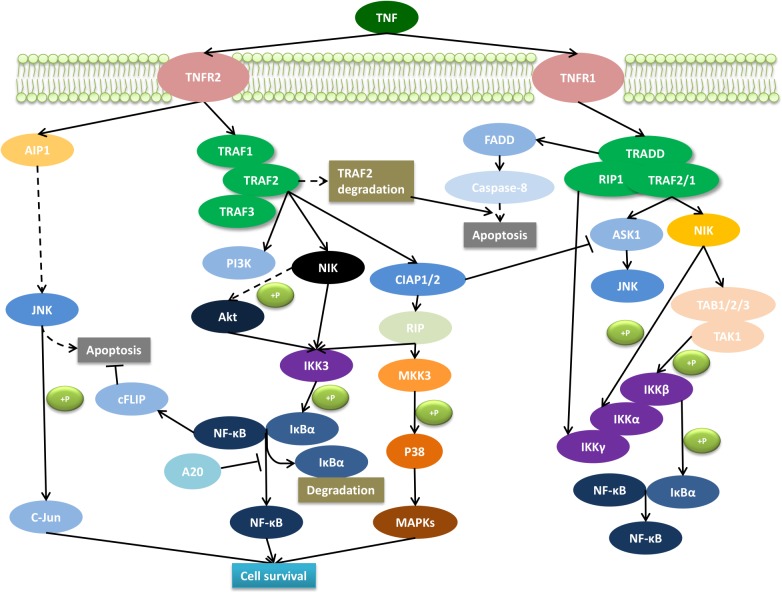
Molecular signaling pathway of TNFR2. The interaction of the membrane form of TNF with TNFR2 promotes cell survival through the activation of the complex signaling cascade. AIP1, ASK interacting protein 1; Akt, serine–threonine kinase; ASK1, apoptosis signal-regulating kinase 1; cFLIP, caspase-8 homolog FLICE-inhibitory protein; cIAP, cellular inhibitor of apoptosis protein; FADD, Fas-associated death domain protein; IKK, IκB kinase; IκBα, inhibitor of kappa B; JNK, c-Jun N-terminal kinase; NFκB, nuclear factor kappa B; MAPKs, phosphorylate mitogen-activated kinase; MKK, mitogen-activated protein kinase kinase; NIK, NFκB-inducing kinase; PI3K, phosphoinositide 3-kinases; RIP, receptor interacting protein; TRADD, TNF receptor-associated death domain; TRAF, TNF receptor-associated factor.

Tumor necrosis factor signaling can elicit various cellular responses through TNFR1 and TNFR2 depending on a variety of factors including the cellular metabolic state and the presence of the adaptor proteins. In fact, the differences in the intracellular signaling pathways such as nuclear factor kappa-B (NF-κB), p38, c-jun N-terminal kinase (JNK), and the ceramide/sphingomyelinase signaling pathway, result in multifarious processes including inflammation, proliferation, cell migration, apoptosis, and necrosis (Eissner et al., 2000, 2004; Harashima et al., 2001; Ware, 2005). TNFR2-mediated signaling activates inflammatory and pro-survival signaling pathways via interaction with adaptor proteins of TRAF1 and TRAF2, and in turn, also the cIAPs and the NF-κB pathway (Rothe et al., 1994, 1995a; Rao et al., 1995) (Figure 1). TNFR2 can also activate the phosphatidyl inositol (PI) 3-kinase/Akt pathway to promote cell survival and proliferation (Marchetti et al., 2004; Dolga et al., 2008; Fischer et al., 2011). In addition, there could be a cross talk between TNFR1 and TNFR2 because of the central role that TRAF2, TRAF1, and cIAP play in both TNFR1 and TNFR2 signaling pathways (Naudé et al., 2011). For instance, it was shown that the depletion of TRAF2 caused by TNFR2 activation accelerates the activation of TNFR1-dependent caspase 8 (Wang et al., 1998; Fotin-Mleczek et al., 2002; Li et al., 2002). In general, TNFRs cross talk is elusive and regulated by both the physiological environment and signaling kinetics between TNFR1 and TNFR2.

## TNF Receptors in Alzheimer’s Disease

Mounting evidence convincingly shows that TNF expression is increased in AD by various methods and means, including genetic (Collins et al., 2000), protein expression (Álvarez et al., 2007) and histological (Zhao et al., 2003) approaches. Considering that TNFR1 exerts inflammatory and pro-apoptotic functions, whereas TNFR2 has a neuroprotective function, it is of utmost importance to define the expression of both receptors in AD. For instance, studies in human post-mortem AD brain tissues have demonstrated that TNFR1 levels are increased while TNFR2 levels are decreased (Zhao et al., 2003; Cheng et al., 2010). Furthermore, Cheng et al. (2010) showed that in the AD brain, TNF had a higher likelihood to bind to TNFR1 than to TNFR2, which might explain the prominent role of TNFR1 in the pathophysiology of AD. Strikingly, deletion of TNFR2 in an AD mouse model exacerbated AD pathology through TNFR1 (Jiang et al., 2014). In this same study, a later overexpression of TNFR2 counteracted the aggravation of the disease (Jiang et al., 2014). This finding reinforces a neuroprotective function of TNFR2 in AD pathology. Furthermore, in a mouse model of acute retinal ischemia, Fontaine et al. (2002) demonstrated that, after inducing retinal damage, mice lacking TNFR1 showed a significant reduction in neurodegeneration while mice lacking TNFR2 showed a significant increase in neurodegeneration. Similarly, deletion of TNFR1 in an AD mouse model resulted in a reduction in the generation of Aβ plaques and an enhancement in learning and memory (He et al., 2007; Olleros et al., 2010), indicating the contribution of TNFR1 to neurodegeneration. Lastly, a recent study demonstrated in AD mouse models that abrogation of TNFR1 results in decreased brain inflammation and in an improvement of amyloidosis and blood–brain barrier integrity (Steeland et al., 2018).

Altogether, these results support the hypothesis that increased expression of TNFR1 aggravates AD pathology, whereas TNFR2 is able to prevent it. Therefore, TNFR1 and TNFR2 represent promising therapeutic targets for the treatment or prevention of AD.

## Targeting TNF and TNFRS as Treatment for Alzheimer’s Disease

Existing therapies that target TNF for clinical use are based on monoclonal antibodies that inhibit its signaling via its receptors. These therapies have been approved for treatment of several inflammatory and autoimmune diseases, including rheumatoid arthritis, psoriatic arthritis or plaque psoriasis. The therapeutic potential of these anti-TNF antibodies has more recently been studied in neurodegenerative diseases. Infliximab is a chimeric IgG1 monoclonal antibody that has a high binding affinity for human TNF. Two similar studies that were performed in AD mouse models showed that intracerebroventricular injection of infliximab reduced tau phosphorylation, TNF levels and Aβ plaques (Shi et al., 2011a), and improved visual recognition memory (Kim et al., 2016). Furthermore, intrathecal treatment of infliximab in a female case with AD showed a significant improvement in cognition (Shi et al., 2011b).

Another biologic anti-TNF drug is etanercept, a combination of the extracellular domain of TNFR2 linked to the Fc portion of a human IgG1. A pilot study conducted in 15 AD patients that received perispinal administration of etanercept resulted in a reduction of cognitive deficits based on the AD Assessment Scale-Cognitive Subscale, the Severe Impairment Battery, and the Mini-Mental State Examination (Tobinick et al., 2006). A follow-up study by the same research group (Tobinick and Gross, 2008) reported a significant cognitive improvement in one AD patient within minutes after perispinal treatment with etanercept. Moreover, treatment of etanercept in an AD mouse model resulted in enhanced cognition as well as a reduction in the hippocampal levels of TNF (Detrait et al., 2014). Additionally, an experiment performed in rats showed that peripheral administration of labeled etanercept is able to penetrate in the CNS (Tobinick et al., 2009), whereas a later similar study in rats reported no penetration of labeled etanercept when injected into the perispinal area (Roerink et al., 2015). These contradicting studies question the mechanism of action of etanercept.

Another limitation of the abovementioned etanercept studies in AD patients is that they did not include a control group receiving placebo and the long-term effects of etanercept were not stated. In fact, Atigari and Healy (2014) described the appearance of psychiatric-like symptoms related to schizophrenia in a patient receiving etanercept with no previous psychiatric history. Furthermore, Butchart et al. (2015) did not find significant differences in cognition nor behavior between AD patients receiving placebo or etanercept in a randomized, placebo-controlled, double-blind study. Nonetheless, they reported a higher prevalence of infections in the etanercept group compared with the placebo group (Butchart et al., 2015). Moreover, Sicotte and Voskuhl (2001) reported that a patient receiving etanercept as treatment for rheumatoid arthritis developed multiple sclerosis and optic neuritis.

The use of a similar biological anti-TNF drug, lenercept, was unsuccessful as treatment for relapsing-remitting multiple sclerosis in a placebo-controlled, double-blind phase 2 study. Indeed, patients receiving lenercept experienced an earlier and significant aggravation of the disease compared with patients receiving placebo (Group, 1999). Other studies have also related TNF blockade with the appearance of autoimmune-like syndromes, like lupus, neuropathies and demyelinating disorders (Prinz, 2011). Finally, Deepak et al. (2013) also claimed a relation between anti-TNF therapies and the development of infections and neurological events.

These studies collectively show that anti-TNF therapies may be disadvantageous, especially in the treatment of neurological diseases. In this respect, total inhibition of TNF might nullify its positive effects, particularly via its TNFR2. Given the evidence described, we will focus on new experimental therapies that selectively interfere with the TNF receptors signaling for the treatment of neurodegenerative diseases:

### Inhibition of TNFR1 by Soluble TNF Inhibitor

As concluded in the previous section, TNFR1 plays a key role in neuroinflammation and apoptosis occurring AD. Since solTNF primarily activates TNFR1, a specific inhibition of solTNF would, in principle, block the functions of TNFR1 without preventing the neuroprotective functions of TNFR2.

**FIGURE 2:**
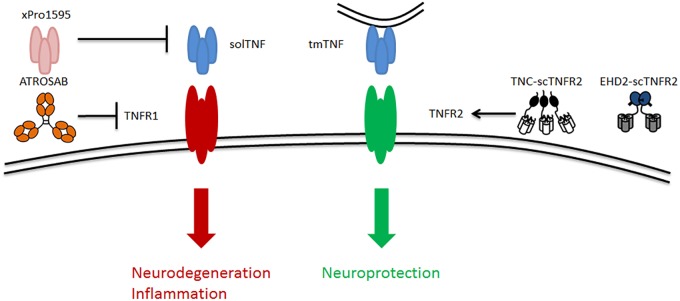
Schematic of TNF signaling via its two receptors TNFR1 and TNFR2 as well as the existing potential therapeutic approaches targeting TNFR2, including solTNF inhibitor [e.g., xPro1595 (Olleros et al., 2010; Shi et al., 2011a)], TNFR1-specific antagonist [e.g., ATROSAB (Kim et al., 2016)], and TNFR2-specific agonists [e.g., TNF-scTNFR2 (Fischer et al., 2011) and EHD2-scTNFR2 (Dong et al., 2016)]. solTNF, soluble tumor necrosis factor; tmTNF, transmembrane tumor necrosis factor.

XPro-1595 is a selective solTNF inhibitor (Figure 2). Results from animal studies show that XPro-1595 improves pathologies in models for neurodegenerative diseases. First, in experimental autoimmune encephalomyelitis (EAE), a multiple sclerosis model, XPro-1595 improved remyelination and reduced CNS lesions and neuroinflammation (Brambilla et al., 2011; Evangelidou et al., 2014). Besides, in a similar study, Taoufik et al. (2011) demonstrated that inhibition of solTNF by XPro-1595 significantly decreased the levels of pro-inflammatory cytokines in the brain and protected mice in the context of EAE. A later study supported the ability of XPro-1595 to ameliorate EAE symptoms in mice, although, in this case, the presence of TNFR2 in oligodendrocytes was necessary (Madsen et al., 2016). This study shows that the solely inhibition of solTNF and, thus, TNFR1 by XPro-1595 might not be sufficient to protect mice from EAE. Moreover, in mice with spinal cord injury, treatment with XPro-1595 significantly increased the protein levels of TNFR2 in the injured area, and enhanced cognition (Novrup et al., 2014). Likewise, a study on acute spinal cord injury in rats that were treated with XPro-1595 showed a significant improvement in immune dysfunction and autonomic dysreflexia symptoms (Mironets et al., 2018). In a mouse model of cerebral ischemia, intravenous administration of XPro-1595 improved motor functions and reduced inflammation (Clausen et al., 2014). Additionally, in a mouse model of Huntington’s disease, intracerebroventricular infusion of XPro-1595 resulted in an improvement of motor functions, increased neuronal viability and decreased neuroinflammation (Hsiao et al., 2014).

Furthermore, in a recent study using a rat model of Parkinson’s disease, peripheral injection of XPro-1595 significantly reduced apoptosis, inflammation, and loss of dopaminergic neurons (Barnum et al., 2014). This study also demonstrated that XPro-1595 can effectively reach the brain by crossing the blood–brain barrier (Barnum et al., 2014). In an AD context, Cavanagh et al. (2016), showed that XPro-1595 was able to prevent synaptic loss when treating mice at an early stage. Finally, MacPherson et al. (2017) showed that systemic administration of XPro-1595 reduced Aβ plaques in the subiculum and restored long-term potentiation in a mouse model of AD. Overall, these findings indicate that XPro-1595 is an effective therapy in disease models where neuroinflammation events are present, suggesting that it could also be used as a potential therapy to treat AD.

### Inhibition of TNFR1 by TNFR1 Antagonist

In addition to inhibition of TNFR1 signaling by removing its most important ligand solTNF, TNFR1 antagonists may serve to inhibit TNFR1 signaling. To this end, Zettlitz et al. (2010) generated an antagonistic TNFR1-specific antibody (ATROSAB), which is able to interfere with the different signaling pathways of TNFR1 (Figure 2). The efficacy of ATROSAB has not yet been tested in AD, but it has been assessed in a nucleus basalis magnocellularis (NBM) chemical lesion model. This *in vivo* model is generated by an exposure to glutamate, which causes neuronal cell death and, mimics acute neurodegenerative diseases. The NBM lesion model provokes an activation of macrophages and microglia (inflammation) and a loss of cholinergic fibers similar to that in AD (Dong et al., 2016). Treatment with ATROSAB or with a TNFR2 agonist (the latter discussed in the section “Stimulation of TNFR2 by TNFR2 Agonist”) reverted these symptoms and protected from memory deficits and excitotoxicity. Besides, by blocking TNFR1, ATROSAB shifted the TNF signaling toward TNFR2, and showed to be neuroprotective in this lesion model (Dong et al., 2016). Importantly, a recent study that tested ATROSAB in the EAE multiple sclerosis model demonstrated that treatment with ATROSAB was able to significantly mitigate EAE symptoms and delay the disease onset, proving the potential efficacy of ATROSAB in this neurodegenerative disease model (Williams et al., 2018). Accordingly, ATROSAB may represent a potential therapy for treating AD.

### Stimulation of TNFR2 by TNFR2 Agonist

Instead of inhibiting TNFR1 signaling in order to prevent cell death, one can promote the signaling through TNFR2 in order to stimulate cell survival. The neuroprotective role of TNFR2 signaling has been reported in several studies (Fontaine et al., 2002; Marchetti et al., 2004; Patel et al., 2012; Maier et al., 2013; Fischer et al., 2014). Hence, Fischer et al. (2011) developed a soluble human TNFR2 agonist (TNC-scTNFR2) that selectively mimics tmTNF, augmenting TNFR2 activation (Figure 2). This agonist proved to protect against neuronal cell death induced by oxidative stress (Fischer et al., 2011), which is a common hallmark of neurodegenerative diseases, including AD. Dong et al. (2016) evaluated the efficacy of another selective TNFR2 agonist (EHD2-scTNFR2) in combination with ATROSAB in the NMB lesion model (Dong et al., 2016). This combination of TNFR1 antagonist and TNFR2 agonist selectively inhibited TNFR1 and enhanced TNFR2 activation, acquiring a potent neuroprotective effect, as revealed by an improvement in memory and cell viability, and a reduction in the loss of cholinergic fibers and inflammation. Overall, this study (Dong et al., 2016) demonstrated that the combination of the antagonistic TNFR1-specific antibody ATROSAB and the selective TNFR2 agonist EHD2-scTNFR2 is effective to treat an acute neurodegenerative disorder caused by glutamate-induced excitotoxicity. Thus, it is plausible that applying this strategy will serve to treat other neurological disorders, like AD.

## Conclusion

The discovery of the apparent dual role of TNF through its two receptors has initiated extensive research into new possibilities to treat neuroinflammation, a common hallmark of neurodegenerative diseases. The initial discovery of anti-TNF therapies led to inconclusive results due to the potential side effects that were reported. Therefore, the development of specific TNFR1 antagonists and solTNF inhibitors (ATROSAB and XPro-1595) that ameliorate inflammation and apoptosis, and TNFR2 agonists that enhance neuro-regeneration and tissue homeostasis, are promising strategies to treat neurodegeneration. As discussed in this mini-review, a considerable number of studies have shown the efficacy of targeting TNF receptors in several neurodegenerative diseases, suggesting that these drugs might have potential in the therapy of AD. In the future, a deeper understanding of the diverse molecular pathways of TNF signaling can contribute to the discovery of more specific and refined strategies to treat AD and other neurodegenerative diseases.

## Author Contributions

NO-C and YW wrote the manuscript. PN, PDD, IZ, and UE reviewed and edited it, and provided key guidance.

## Conflict of Interest Statement

The authors declare that the research was conducted in the absence of any commercial or financial relationships that could be construed as a potential conflict of interest.
